# Nursing interventions to ensure the safety of critically ill patients in intrahospital transportation: A scoping review

**DOI:** 10.1177/17511437261422929

**Published:** 2026-04-06

**Authors:** Ana Filipa Lemos, Diana Vareta

**Affiliations:** 1 Department of Nursing, Arco Ribeirinho Local Health Unit, Barreiro, Portugal; 2 Nurs* Lab, Egas Moniz School of Health & Science, Caparica, Caparica, Almada, Portugal; 3 Egas Moniz Center for Interdisciplinary Research (CiiEM), Egas Moniz School of Health & Science, Caparica, Almada, Portugal

**Keywords:** intrahospital transportation, critically ill patients, patient safety, nursing care

## Abstract

**Background:**

Intrahospital transportation of critically ill patients is a high-risk process, frequently linked to complications and increased vulnerability to adverse events. Ensuring safety requires nursing interventions that uphold the standards of care provided in critical care units.

**Objective:**

To identify nursing interventions that promote patient safety during intrahospital transportation. Methods: A scoping review was conducted following Arksey and O’Malley’s framework. The search was performed in 2024 across PubMed and EBSCOhost (CINAHL Complete, MEDLINE Complete, Nursing & Allied Health Collection: Comprehensive, and Cochrane Central Register of Controlled Trials).

**Results:**

Seven publications met the criteria. All studies emphasized the need for strategies to prevent complications during transport. Four categories of interventions emerged: identification and management of adverse events, use of checklists, continuous nurse training, and effective communication.

**Conclusions:**

Nurses play a pivotal role in maintaining safety and quality of care. By applying evidence-based strategies, they minimize risks and ensure safer intrahospital transportation.

## Introduction

According to the World Health Organization (WHO),^1^ patient safety is defined as reducing the risk of unnecessary healthcare harm to the lowest acceptable level. The main point is to focus on the absence of harm that healthcare produces compared to the benefit of healthcare itself.

Safety incidents in healthcare continue to be a global concern. The World Health Organization’s Global Patient Safety Action Plan 2021–2030^1^ outlines seven strategic objectives aimed at achieving the greatest possible reduction in avoidable harm caused by unsafe care. Within this framework, nurses, as key providers of patient care, have a fundamental responsibility to implement and adapt these strategies in clinical practice, thereby enhancing the safety and quality of care delivered.

The critically ill patient is at risk of organ or system dysfunction or failure, requiring advanced monitoring and therapeutic measures to maintain physiological stability and survival.^2,3^ However, when these patients undergo transportation to areas where emergency response capabilities are inadequate, their vulnerability increases significantly.^4,5^ The absence of immediate access to life-saving interventions during transport can further exacerbate an already unstable condition, underscoring the importance of rigorous safety measures and thorough preparedness in such situations to avoid complications.^4,6^

The transportation of a critically ill patient can be divided into primary and secondary transport. Primary transport corresponds to pre-hospital transport to a health unit, while secondary transport includes transport between healthcare units.^7,8^ It is important to note that critically ill patients are often subjected to intrahospital transportation due to the need to carry out complementary diagnostic exams, therapeutic procedures, or transfer to the operating theater or intensive care unit.^1^ The existence of intrahospital transportation, which is not included in the two previous categories and consists of the transport of critically ill patients within the healthcare institution.^8^

An intrahospital transport team typically is composed of a critical care nurse and a physician or anesthesiologist, depending on the patient’s condition and level of care required, all with experience in advanced life support and specific training for critically ill patient transportation.^5,8^

Intrahospital transportation comprises three phases: decision, planning and implementation.^8^ The first phase of the decision to transport a critically ill patient is the sole responsibility of the physician, in agreement with the team leader, and involves a careful assessment of both patient-specific and transport-related risks. Planning is carried out jointly by the medical and nursing team. It includes contacting the destination service, estimating distance and travel time, selecting the transport team, and defining the necessary monitoring and physiological targets to be maintained throughout the transfer. It also encompasses the selection of equipment and therapy, as well as the anticipation of potential complications. The transport team is responsible for carrying out the intrahospital transportation, ensuring that the patient is monitored until they are handed over to the receiving service, or in the case of complementary diagnostic tests or other therapeutic acts until the patient returns to the service of origin.^5,8,9^

The transport of critically ill patients is associated with significant complications and frequent adverse events, most commonly involving respiratory and hemodynamic instability, accidental dislodgement of life-support equipment such as endotracheal tubes, venous accesses, oxygen supply, malfunction or failure of the transport ventilator, exteriorization or clamping of drains, and interruptions in vital monitoring.^4,6,10,11^ The transfer to the stretcher, the first 5 min of transport, and prolonged transport (up to 30 min) are mentioned as the moments of most significant risk of adverse events.^5^

To reduce the associated risks, transportation must be organized and accompanied by adequate monitoring, equipment, and supervision. The level of monitoring, surveillance, and care during transport must be at least equal to that of the service of origin, and the failure to prepare either the patient or the transport team can decrease the rigor of the care provided to the patient.^8^

Nurses play a pivotal role in< safeguarding the quality and effectiveness of intrahospital transport for critically ill patients. Given the inherent instability of these patients and the constant risk of deterioration affecting one or more vital functions, transport must occur under conditions that guarantee safety and allow for early anticipation and prompt detection of complications.^6,10^ Despite the high prevalence of adverse events reported in the literature, evidence on the specific contributions of nurses in mitigating these risks remains fragmented. This review seeks to identify and synthesize nursing interventions that promote the safety of critically ill patients during intrahospital transport, thereby contributing to the development of evidence-based practices and improving patient outcomes.

### New contribution

This scoping review consolidates fragmented evidence on nursing interventions for the intrahospital transport of critically ill patients, outlining four key domains: adverse event identification, checklist use, nurse training, and effective communication. By mapping these interventions into a practical framework and highlighting gaps in practice and in the use of simulation-based training, the study advances evidence-based nursing practice and supports the development of safer, standardized protocols.

## Materials and methods

The scoping review was conducted according to Arksey and O’Malley’s methodological framework,^12^ which comprises five stages: (1) identifying the research question, (2) identifying relevant studies, (3) selecting studies, (4) charting the data, and (5) collating, summarizing, and reporting the results. To strengthen clarity, maintain transparency, and ensure methodological rigor, the Preferred Reporting Items for Systematic Reviews and Meta-Analyses extension for Scoping Reviews (PRISMA-ScR) checklist was applied.^13^

### Stage 1: Identifying research question

The research question was developed following the recommendations of the Joanne Briggs Institute PCC mnemonic^14^ which is based on the Population (P), Concept (C), and Context (C) components. provides a systematic approach for defining the scope and boundaries of a review by clearly identifying its key elements.

Accordingly, the research question guiding this review was: *What nursing interventions ensure the safety of critically ill patients during intrahospital transportation?*

### Stage 2: Identifying relevant studies

The literature search was conducted in the scientific databases PubMed and EBSCOhost, including CINAHL Complete, MEDLINE Complete, Nursing & Allied Health Collection: Comprehensive, and the Cochrane Central Register of Controlled Trials. A comprehensive search strategy was developed in accordance with the PCC framework, combining key concepts mapped to Medical Subject Headings (MeSH) and Health Sciences Descriptors. Boolean operators “AND” and “OR” were applied to structure the search, resulting in the following equation: [(critically ill patient OR critical illness) AND (patient safety) AND (patient transfer OR intrahospital transport)].

The inclusion criteria established to address the research question were:

Population: Patients in critical condition;
Concept: Nursing interventions aimed at ensuring safety;
Context: Intrahospital transportation.

The review was conducted in January 2024 and encompassed qualitative, quantitative, mixed-methods studies, as well as literature reviews addressing nursing care to ensure the safety of critically ill patients during intrahospital transport. Eligible studies were published between 2018 and January 2024, written in English, Portuguese, or Spanish, and available in full text. Two researchers independently carried out the search, study selection, and data extraction, with all records that did not meet the inclusion criteria being excluded.

### Stage 3: Study selection

After removing duplicates, the titles and abstracts of the remaining records were screened according to the predefined selection criteria. Full-text articles deemed potentially relevant were then assessed in detail, and those meeting the eligibility criteria were included in the review. In cases of uncertainty, articles were advanced to the next stage, and any disagreements between reviewers were resolved by consultation with a third reviewer.

**Figure 1.**
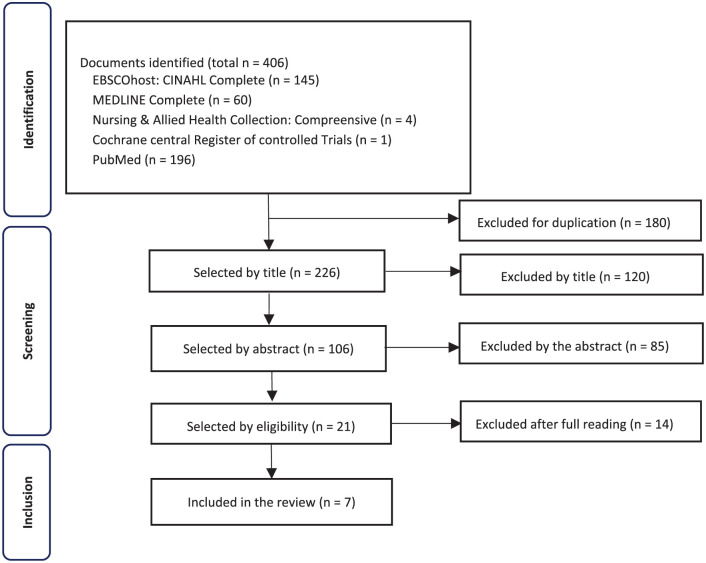
PRISMA flow chart for study selection.

The search initially yielded 406 records across the following databases: CINAHL Complete (*n* = 145), MEDLINE Complete (*n* = 60), Nursing & Allied Health Collection: Comprehensive (*n* = 4), Cochrane Central Register of Controlled Trials (*n* = 1), and PubMed (*n* = 196). After removing duplicates (*n* = 180), 226 titles and 106 abstracts were screened, followed by 21 full-text assessments. Ultimately, seven articles met the eligibility criteria and were included in this review. The PRISMA flowchart demonstrates the identification, screening and selection process of studies (Figure 1).

### Stage 4: Charting the data

Data extraction was carried out independently by two reviewers using a tool specifically developed to address the research question of this review. The instrument was designed to guarantee the systematic and comprehensive capture of relevant information.^12^ Extracted items included general study details (author, year of publication, title, and country), which provided contextual background for the analysis. Methodological characteristics, such as study design and objectives, were also recorded. Additionally, nursing interventions data were collected, with a particular focus on the strategies to ensure transport safety. The completed data chart was subsequently reviewed and discussed collectively by all authors to ensure accuracy, relevance, and consistency, thereby strengthening the rigor and validity of the review.

### Stage 5: Collecting, summarizing and reporting the results

To address the research question, the scientific articles included were analyzed and synthesized in a table (Table 1) containing title, author, year and country of publication, study objective, methodology, and the main interventions or phenomena of interest. A thematic analysis, guided by Braun, Clarke, Hayfield, and Terry’s^15^ framework, was used to organize and interpret the data. Two researchers independently performed manual coding to identify recurring themes across the studies.

**Table 1.** Data extraction and synthesis.

Authors/Year/Country/Title	Aim	Study design	Nursing interventions
O’Leary et al.^17^ 2018, Iran Transfer of critically ill adults – assessing the need for training	Explore organizational, environmental and individual issues that increase patient risk during intrahospital transportation	Qualitative descriptive study	• Practical training for nurses • Training nurses through simulated practice
Akrami et al.^16^ 2019, Iran Assessing the Effect of Training the Safe Transfer Checklist on the Quality of Intrahospital Patient Transfer: An Interventional Study	To determine the impact of nurses’ training in the use of checklists on the quality of intrahospital transportation	Quasi-experimental study	• Implementation of an IHT checklist • Practical training for nurses
Sharafi et al.^18^ 2020, Iran Improving the safety and quality of the intra-hospital transport of critically ill patients	Determine the incidence of adverse events during intrahospital transportation and obtain suggestions from nurses to improve this process.	Cross-sectional study	• A patient-centered approach to intrahospital transportation: focusing on the patient’s clinical condition (continuous and personalized monitoring) and involving the family in the transfer process, as this reduces the patient’s anxiety, increases their satisfaction and improves the quality of care provided. • Promoting effective communication between nurses • Practical training for nurses
Williams et al.^19^ 2019, Australia A checklist for intrahospital transport of critically ill patients improves compliance with transportation safety guidelines	Assess compliance with intrahospital transportation guidelines before and after applying a transportation checklist	Prospective, pre- and post-interventional study	• Training nurses on the intrahospital transportation checklist • Application of intrahospital transportation checklist • Improved communication with the use of a checklist
Putra et al.^21^ 2022, Indonesia Adverse events during intra-hospital transport of critically ill patients: an observational study	Describe adverse events in transportation between the emergency room and the intensive care unit	Cohort study	• Practical training for nurses • Implementation of intrahospital transportation checklist
Nisha & Sariga.^20^ 2022, India Risk Factors and Adverse Events During Intra Hospital Transportation Among Critically Ill with a View to Develop Patient Transport Checklist	Determine risk factors associated with intrahospital transportation, adverse events. Develop an intrahospital transportation checklist	Descriptive study	• Development of an IHT checklist considering the risk factors and adverse effects found in the study
Hu et al.^22^ 2021, China Intrahospital transport of critically ill patients: A survey of emergency nurses China	Investigate nurses’ knowledge and perception of intrahospital transportation; understand the relationship between established protocols and adverse events	Retrospective study	• Implement practical training courses for nurses • Periodic assessment of nurses’ knowledge of IHT • Debriefing on adverse effects during IHT to build coping strategies • Develop and implement intrahospital transportation checklists/protocols • Emphasize communication between the nurses of the originating service and the destination service

## Results

Seven articles met the illegibility criteria and were incorporated in the review at the end of the selection process. They were conducted across diverse settings: three in Iran^16–18^ and one each in Australia^19^ Indonesia,^20^ India,^21^ and China.^22^ Methodological designs varied, comprising two descriptive studies,^16,21^ a prospective study,^19^ a quasi-experimental study,^17^ a cohort study^20^, a cross-sectional study,^18^ and a retrospective study.^22^

As presented in Table 1, several nursing interventions were identified as key strategies to promote the safety of critically ill patients during intrahospital transport.

These interventions were grouped into four categories: Identification of potential adverse events, checklist implementation, nurse training and effective communication. Each category is described below:

### Identification of potential adverse events

Most authors highlight equipment-related problems as the most common adverse events.^18,20–22^

Nisha and Sariga^20^ reported that 43% of events were linked to equipment malfunction and 17% to monitoring failures. Putra et al.^21^ found that 78.8% of patients transferred from the emergency department to the intensive care unit experienced adverse events, the majority associated with inadequate equipment preparation. Sharafi et al.^18^ identified psychomotor agitation, dislodgement of intravenous lines, and oxygen desaturation as the most frequent complications across all transport phases, recommending rigorous checks of both equipment functionality and oxygen supply before transfer. Similarly, Hu et al.^22^ observed that 51.1% of incidents involved nasogastric tube dislodgement, 31.8% orotracheal tube removal, 44.6% oxygen supply issues, 44.6% monitor malfunction, and 62.7% removal of peripheral venous catheters.

### Checklist implementation

Six of the seven studies highlighted checklists as a key strategy to standardize procedures, enhance safety, and minimize errors during intrahospital transport.^16,17,19–22^ Their use helps ensure that essential resources are available and that all critical steps are consistently verified before, during, and after transfer. Williams et al.^19^ reported improved compliance with Australian and New Zealand of Anaesthetists guidelines, increasing from 86% to 90% following checklist implementation. They also noted that checklists guaranteed 100% accuracy in patient identification and equipment preparation, while strengthening communication with destination teams. Similarly, Akrami et al.^16^ demonstrated that checklist use improved the overall quality of intrahospital transport.

### Nurse training

The safe transport of critically ill patients requires transport teams to assess individual risks, anticipate complications, and respond effectively during transit. Six studies stressed the importance of nurse training to strengthen technical skills in patient monitoring, equipment handling, documentation, and communication.^16–22^

Hu et al.^22^ found that although 96.2% of nurses acknowledged the risk of severe complications or death during intrahospital transport, only 70.1% had received specific training, underscoring the need for regular knowledge assessments and ongoing education. While most authors focused on practical training, only O’Leary et al.^17^ emphasized the value of simulation-based training as a means of improving practice and ensuring greater patient safety during transport.

### Effective communication

Effective communication was one of the most frequently cited aspects across studies. Prior coordination between the transport nurse and the destination service was seen as essential to ensure resource availability, service readiness, and continuity of care. Sharafi et al.^18^ emphasized collaboration between transport teams, physicians, and destination staff as crucial for minimizing risks. Communication with the critical patient was also highlighted as an important safety measure, since explaining the procedure and encouraging cooperation helped reduce adverse events.

According to Williams et al.,^19^ the use of checklists further improved communication with destination services, ensuring that teams were adequately prepared to receive critically ill patients.

## Discussion

The findings of this review identified four central domains of nursing intervention during the intrahospital transport of critically ill patients: Identification of potential adverse events, use of checklists, continuous training of nurses, and effective communication between transport and destination teams.

Several studies have confirmed the high frequency of adverse events, most of which are linked to equipment malfunction, dislodgement of medical devices, or hemodynamic instability.^17–22^ These results align with international recommendations, which consistently emphasize that incidents such as accidental extubation, dislodgement of venous access, inadequate oxygen supply, ventilator malfunction, and failures in equipment batteries remain major threats during transport.^7^ Preventive strategies, such as systematic equipment checks and rigorous preparation before transfer, are therefore indispensable.

The evidence strongly supports the use of checklists as an effective safety tool. Studies have demonstrated that their implementation reduces complications, improves compliance with clinical guidelines, ensures accurate patient identification and equipment preparation, and facilitates better communication with receiving units.^17,23,24^ In addition, checklists contribute to standardizing procedures, reducing variability in practice, and minimizing preventable risks.^7,10,25^

Training emerged as another crucial element. Nurses are often the most directly responsible for detecting, preventing, and managing adverse events during transport.^26^ However, research shows that a significant proportion of nurses have not received specific preparation for intrahospital transport.^16,21^ Simulation-based education has been highlighted as a particularly valuable approach, enabling teams to develop both technical and non-technical skills, such as communication, coordination, decision-making, and situational awareness.^16,23,26^ Continuous professional development, including Advanced Life Support and Critical Patient Transport training, is essential for maintaining competence and ensuring readiness.^7,8^ Effective communication is also a cornerstone of safe transport. Pre-transport coordination with the destination unit allows teams to anticipate needs, prepare resources, and secure the availability of specialized staff.^27^ Communication with the patient, when possible, has also been shown to reduce anxiety and foster cooperation, which can help prevent complications.^25^ Checklists further reinforce this process by prompting systematic information exchange among teams.^16,18,19,22^

Overall, the success of intrahospital transport depends on the integration of these elements. A skilled team must conduct transport, ideally composed of at least two professionals with expertise in advanced life support and specific training in the transfer of critically ill patients.^27^ Beyond technical proficiency, non-technical competencies, including leadership, teamwork, and coordination, are equally decisive in maintaining patient safety and continuity of care throughout the transfer.^26^

Despite the consistency of these findings, the evidence base remains limited. Only a small number of studies specifically addressed intrahospital transport, and none were conducted in American or European contexts, which restricts the ability to draw context-sensitive conclusions. The scarcity of primary research in this field and the concentration of studies in non-occidental healthcare systems highlight the need for further investigations that reflect the realities of different organizational and cultural contexts. Expanding the evidence base would allow for more comprehensive and transferable recommendations to strengthen nursing practice and patient safety during intrahospital transport.

## Conclusions

The intrahospital transport of critically ill patients is a frequent yet high-risk procedure in hospital settings, where nurses assume a central role in safeguarding patient safety. Although transfers occur within the hospital, the evidence consistently demonstrates a high incidence of adverse events, underscoring the need for rigorous planning and preventive measures. Careful patient assessment, anticipation of potential complications, and thorough preparation of resources are fundamental strategies to minimize risks and promote safety throughout the process.

The studies reviewed converge on the importance of adopting structured interventions, particularly the use of checklists, continuous monitoring, route assessment, equipment preparation, effective communication, and targeted nurse training. These measures not only reduce the likelihood of adverse events but also strengthen the quality and continuity of care during transport.

Nurses are uniquely positioned to lead these safety practices, as they are responsible for ensuring that the care provided during transport is equivalent to, or even exceeds, the standard delivered in the original unit. By combining technical expertise with non-technical competencies such as coordination, communication, and situational awareness, nurses can significantly improve outcomes for critically ill patients during intrahospital transport.

Despite these insights, research in this area remains scarce, particularly within European contexts, highlighting the need for further studies to evaluate the impact of nursing interventions on patient outcomes and to inform the development of evidence-based protocols. Expanding the knowledge base will be essential to advancing safe, high-quality care in this critical component of hospital practice.
